# Robotic Assist and Virtual Surgical Planning in Orthognathic Surgery

**DOI:** 10.31661/gmj.v13iSP1.3672

**Published:** 2024-12-08

**Authors:** Mehdi Abrishami, Fatemeh Pourabdolah, Behnaz Dalvandi, Reza Dalvandi, Negar Sarrafan, Sara Noorizadeh, Alireza Etezadinia, Sahar Negargar

**Affiliations:** ^1^ Department of Oral and Maxillofacial Surgery, Isfahan (Khorasgan) Branch, Islamic Azad University, Isfahan, Iran; ^2^ Department of Psychiatry, School of Midwifery Nursing, Mazandaran University of Medical Sciences, Sari, Iran; ^3^ The Islamic Republic of Iran Medical Council, Tehran, Iran; ^4^ Department of Oral and Maxillofacial Medicine, School of Dentistry, Urmia University of Medical Sciences, Urmia, Iran; ^5^ Department of Periodontics, Faculty of Dentistry, Shahed University, Tehran, Iran; ^6^ Department of Periodontology, Shahed University, Tehran, Iran; ^7^ Department of Dentistry Asad Abadi Hospital Tabriz, Iran

**Keywords:** Virtual Surgical Planning (VSP), Robotic Assistance, Orthognathic Surgery, Surgical Precision

## Abstract

Orthognathic surgery, critical for correcting jaw deformities and improving facial function and aesthetics, has undergone transformative changes with the introduction of robotic assistance and Virtual Surgical Planning (VSP). These technologies have revolutionized the field by enhancing precision, reducing operative times, and enabling more predictable surgical outcomes. Robotic systems, including the Da Vinci® and ROSA® platforms, provide sub-millimeter precision in osteotomies, while VSP enables comprehensive preoperative planning by integrating advanced 3D imaging and simulation techniques. Together, these technologies provide an unparalleled level of control and precision in surgical procedures, significantly enhancing patient outcomes. Major advancements in the field include the integration of artificial intelligence and machine learning into surgical planning, which allows for better prediction of postoperative outcomes and real-time adjustments during surgery. Augmented reality is also gaining traction as a tool for intraoperative guidance, further enhancing the precision of robotic-assisted procedures. Emerging technologies such as haptic feedback systems and next-generation robotic arms hold promise for even greater improvements in surgical accuracy and efficiency. The relevance of these technologies to clinical practice is profound. By reducing complications, enhancing accuracy, and improving both functional and aesthetic results, robotic assistance and VSP are redefining standards in orthognathic surgery. However, barriers related to cost, surgeon training, and infrastructure must be addressed to enable the widespread adoption of these technologies. Future research should focus on validating these technologies in large-scale clinical trials and assessing their long-term benefits and cost-effectiveness. Ultimately, the integration of these cutting-edge technologies has the potential to revolutionize orthognathic surgery, making it safer, more efficient, and more personalized for patients.

## Introduction

**Figure-1:**
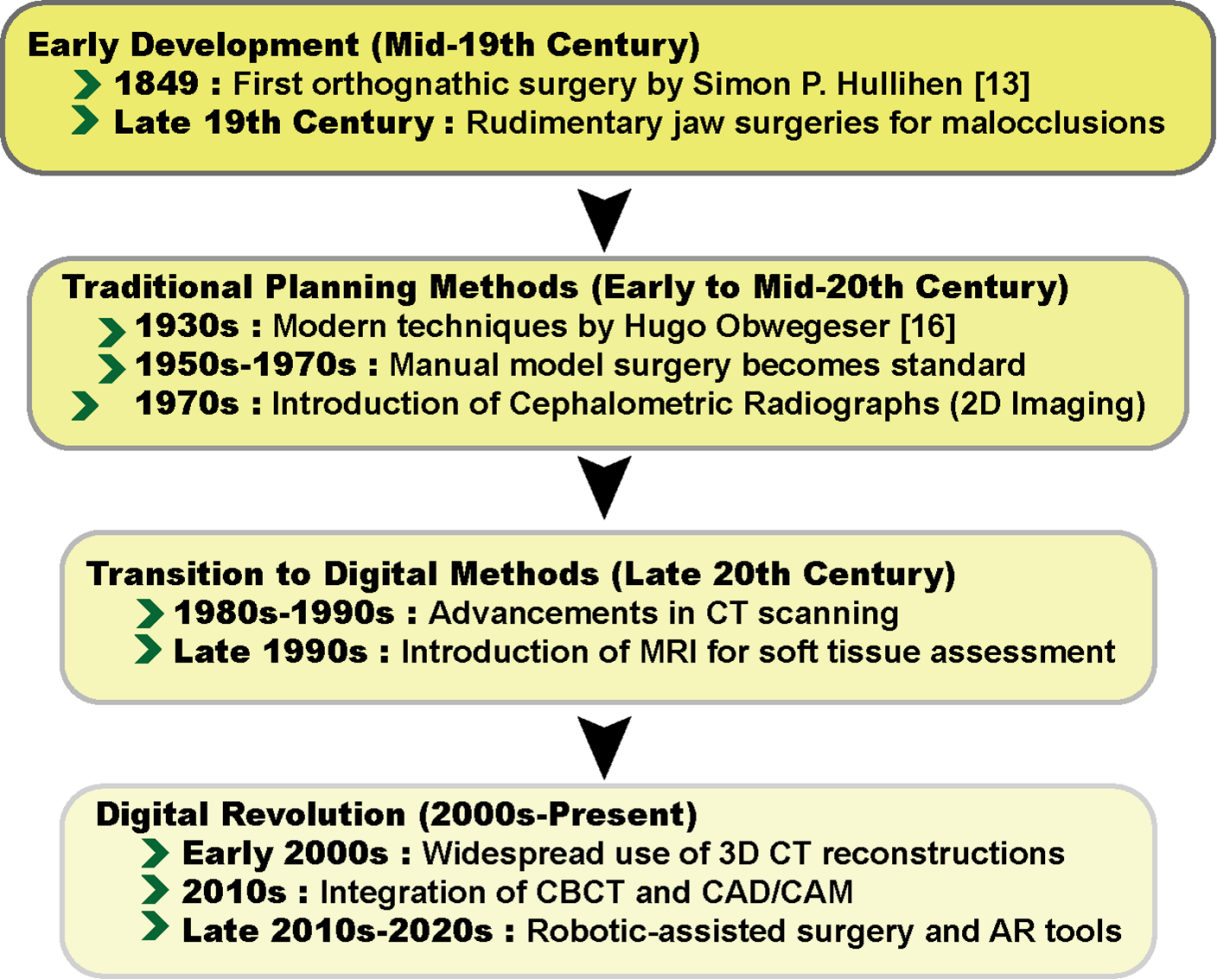
Figure 1. Key Milestones in the Historical Development of Orthognathic Surgery.

**Table:** Table1. Comparison of Clinical Outcomes Between VSP and Conventional Techniques

**Metrics**	**VSP**	**Conventional Techniques**	**Reference**
**Operative Time (hours)**	2.5 - 4 hours	3 - 9 hours	[39][40]
**Accuracy (mm deviation)**	<1 mm	>1 mm	[34]
**Complication Rates**	Low (<5%)	Higher (up to 15%)	[41]

Orthognathic surgery, a cornerstone in the correction of jaw deformities, has undergone significant advancements in recent decades [1]. Traditionally, these surgeries relied heavily on surgeon experience and manual dexterity, requiring extensive planning, complex intraoperative maneuvers, and posing challenges in ensuring precise outcomes [2]. As a result, surgical outcomes varied, with issues such as prolonged operating times, increased risk of complications, and inconsistencies in jaw alignment frequently reported [3]. These limitations have spurred the adoption of cutting-edge technologies aimed at improving precision, efficiency, and predictability in orthognathic procedures [4]. Two of the most transformative innovations in this field are robotic assistance and VSP [5]. Robotic-assisted surgery, once predominantly utilized in fields like urology and general surgery, has found increasing applications in maxillofacial surgery [4]. Robotic systems, such as the Da Vinci® Surgical System and ROSA®, have transformed complex jaw realignment surgeries by enhancing precision and minimizing human error [6]. These systems enable surgeons to execute highly accurate bone cuts and ensure optimal repositioning of the mandible and maxilla, ultimately leading to improved clinical outcomes and patient satisfaction [4].

Simultaneously, the advent of VSP has introduced a paradigm shift in preoperative preparation [2]. VSP integrates advanced imaging modalities, including three-dimensional (3D) computed tomography (CT) scans and computer-aided design/computer-aided manufacturing (CAD/CAM) technologies, to create patient-specific surgical models [7]. Through simulation of the surgical procedure, VSP allows surgeons to precisely plan osteotomies, determine optimal jaw positioning, and anticipate potential challenges before entering the operating room [8]. This personalized approach enhances accuracy, reduces intraoperative risks, and shortens operative times [9].

While both robotic assistance and VSP individually offer significant benefits, recent developments have demonstrated the potential of integrating these technologies to further improve outcomes in orthognathic surgery [4]. When used in synergy, robotic systems, guided by detailed preoperative virtual plans, offer unprecedented precision in executing complex surgical movements [10]. This combination has shown promising results in terms of reducing complications, improving jaw symmetry, and enhancing overall surgical outcomes [4].

Despite these advancements, challenges remain. The cost of acquiring and maintaining robotic systems, the steep learning curve for surgeons, and the need for advanced training in VSP represent barriers to widespread adoption [11]. Furthermore, questions about the comparative effectiveness of these technologies relative to traditional methods warrant deeper investigation [3].

This review primarily aims to explore the synergy between robotic assistance and VSP in orthognathic surgery, evaluating their combined clinical efficacy in comparison to traditional methods and their potential to reshape the future of surgical practices.

Historical Development of Orthognathic Surgery

Orthognathic surgery, derived from the Greek terms "orthos" (meaning straight) and "gnathos" (meaning jaw), has experienced considerable evolution since its inception [12]. This specialized field has advanced significantly due to the continuous development of surgical techniques, imaging technologies, and treatment planning methods [13]. While initially dependent on manual and analog approaches, the field has seen a substantial shift towards digital technologies in recent decades, transforming both the planning and execution of surgical procedures (Figure-1) [8]. illustrates the key milestones in the evolution of orthognathic surgery.

The origins of orthognathic surgery can be traced to the late 19th and early 20th centuries when surgeons began performing corrective jaw surgeries to address significant maxillofacial deformities [14]. In the absence of sophisticated imaging and planning tools, surgeons primarily relied on manual model surgery [15]. This involved creating physical dental casts of the patient’s upper and lower jaws, which were then cut and repositioned to simulate the surgical movements that would be performed intraoperatively to correct skeletal misalignments [16].

By the mid-20th century, the advent of two-dimensional (2D) imaging, particularly lateral and frontal cephalometric radiographs, represented a major advancement in the field [15][17]. These radiographs enabled surgeons to evaluate skeletal discrepancies and facial proportions and to plan surgical interventions [9]. However, 2D imaging had limitations in fully capturing the three-dimensional (3D) complexity of craniofacial anatomy and in predicting soft tissue changes following surgery, particularly in cases involving more complex deformities [7].

A pivotal period for the field came in the 1980s and 1990s with the introduction of 3D imaging technologies [15]. Computed tomography (CT) and, later, cone beam computed tomography (CBCT) provided detailed cross-sectional images of craniofacial structures, allowing surgeons to visualize both bony and soft tissue anatomy in three dimensions [18]. This advancement enabled more accurate diagnoses and enhanced treatment planning, significantly improving the precision of surgical outcomes [19].

Parallel to the advancements in imaging, computerized surgical planning systems emerged during this period [20]. Early digital systems allowed surgeons to virtually manipulate 3D models of the patient’s jaws and skulls, simulating osteotomies and enabling more accurate predictions of postoperative outcomes [6]. These early systems laid the groundwork for what would later become known as VSP, which has since revolutionized orthognathic surgery [21].

By the late 1990s and early 2000s, robotic systems and VSP further transformed the field. VSP, utilizing advanced 3D imaging, computer-aided design (CAD), and computer-aided manufacturing (CAM), marked a significant leap forward, enabling real-time adjustments to skeletal movements and providing more accurate predictions of soft tissue changes, thereby improving both functional and aesthetic outcomes [15].

Robotic systems, initially developed for other surgical disciplines such as urology and cardiac surgery, began to be incorporated into maxillofacial surgery in the early 2000s [22]. One of the first robotic systems to be applied in orthognathic surgery was the Da Vinci® Surgical System [23]. Initially developed in the late 1990s, its application in maxillofacial procedures in the early 2000s improved the precision of bone cutting and suturing [24]. Developed later, the ROSA® system was initially designed for neurological procedures but was subsequently adapted for craniofacial surgeries [25]. This system allows for robotic-guided osteotomies with increased accuracy and has demonstrated promising results in terms of precision and reduced operating times [26]. While robotic assistance was being explored to enhance the execution of surgical procedures, VSP continued to evolve, offering substantial advancements in the preoperative phase [9].

Early 2000s: The first practical VSP systems enabled surgeons to manipulate 3D models based on CT scans and design cutting guides. However, these early systems required manual adjustments during surgery due to limitations in computational power and software [10].

Mid-2000s: With the integration of Computer-Aided Surgical Simulation (CASS), VSP became more sophisticated, allowing surgeons to simulate the precise repositioning of jaw segments and predict both aesthetic and functional outcomes of surgery [2].

Late 2000s to Early 2010s: The incorporation of 3D printing technology allowed for the production of patient-specific surgical guides and splints, which could be used intraoperatively to guide osteotomies with millimeter-level precision [7]. As both robotic systems and VSP evolved, their integration became a natural next step [4]. By integrating preoperative virtual plans with robotic execution systems, surgeons can achieve unprecedented levels of precision during surgery. Robotic systems, guided by the detailed instructions generated through VSP software, can execute preplanned osteotomies with extraordinary accuracy, minimizing human error [6]. This synergy is being explored in recent clinical trials and studies, showing encouraging results in terms of reduced operative time, improved aesthetic outcomes, and enhanced patient safety [10].

Current State of Robotic Assist in Orthognathic Surgery

Robotic assistance in orthognathic surgery has emerged as a promising solution to improve precision, reduce variability in surgical outcomes, and enhance the overall safety of procedures [5]. The development of sophisticated robotic platforms, such as the Da Vinci® Surgical System and the ROSA® robotic system, has provided surgeons with tools to carry out delicate and complex maxillofacial procedures with unprecedented accuracy [27]. These robotic systems are transforming the field, offering significant advantages in terms of control, precision, and patient outcomes [24].

Technologies Used

Two of the most prominent robotic platforms utilized in orthognathic surgery are the Da Vinci® Surgical System and the ROSA® robotic system. The Da Vinci® system has found its way into orthognathic surgeries due to its ability to facilitate precise surgical movements [28]. The system employs robotic arms equipped with micro-instruments, which are controlled by the surgeon from a console. The high-definition, 3D visualization provided by the system enhances the surgeon’s ability to perform precise osteotomies and bone repositioning with minimal tremor and maximal control [29]. The wristed instruments offer a range of motion greater than the human hand, allowing for intricate movements that are difficult to achieve through conventional methods. Studies have shown that the use of Da Vinci® in orthognathic procedures can result in improved precision during bone cutting and fixation, reducing the risk of human error [30].

The ROSA® system, originally developed for neurosurgery, has been adapted for use in maxillofacial and orthognathic surgeries due to its versatility and ability to assist in both bone cutting and positioning [31]. ROSA® employs robotic arms with high levels of spatial accuracy, which can execute pre-programmed surgical plans designed through VSP. Unlike Da Vinci®, which focuses on enhancing the surgeon’s manual dexterity, ROSA® provides automated or semi-automated functions that reduce the burden on the surgeon during critical stages of the surgery, such as osteotomy and maxillary repositioning [32]. ROSA® also integrates with imaging systems to provide real-time feedback, allowing for adjustments during the surgery to ensure optimal alignment of the jaw [5].

Clinical Applications

Robotic systems have a variety of clinical applications in orthognathic surgery, particularly in enhancing the precision and efficiency of tAhe procedure. The high surgical accuracy of this system, especially in osteotomies where precision is critical to achieving desired outcomes in jaw alignment [27][33]. By following pre-planned paths based on VSP, robotic systems can cut bone with sub-millimeter accuracy, which is difficult to achieve with manual techniques [5][34]. This level of precision is crucial for avoiding postoperative complications, such as malocclusion or asymmetry [32]. In addition, robotic systems have been shown to significantly reduce operating times. The automation of certain surgical tasks, such as bone cutting and plate fixation, accelerates the procedure and minimizes the time patients spend under anesthesia [24]. This is particularly beneficial in complex cases that require intricate adjustments to the mandible or maxilla, where manual procedures may be time-consuming and prone to error [5]. Another significant clinical advantage is the improvement in patient outcomes, including faster recovery times, fewer complications, and enhanced aesthetic results [5]. By achieving greater precision in bone realignment, robotic systems help ensure that patients experience fewer postoperative issues, such as jaw misalignment or the need for revision surgeries [27]. The robotic system is a new technology and there is limited clinical trials to compare this technology to traditional methods in orthognathic surgery.

VSP in Orthognathic Surgery

VSP has become an integral part of modern orthognathic surgery, enabling surgeons to plan complex jaw corrections with greater accuracy and confidence [35]. By integrating advanced imaging, software tools, and simulation techniques, VSP significantly enhances the precision of surgical procedures, reduces intraoperative risks, and improves patient outcomes [36]. The development of sophisticated software along with 3D imaging and CAD/CAM technologies, has revolutionized the way surgeons approach preoperative planning and execution of orthognathic surgeries [37].

Techniques and Software

VSP involves a combination of 3D imaging techniques and advanced software tools to simulate surgical procedures before entering the operating room [35]. The process typically begins with the acquisition of detailed CT scans or CBCT (Cone Beam Computed Tomography) images of the patient's craniofacial structures. These high-resolution 3D images provide a comprehensive view of the bones, soft tissues, and teeth, which are essential for accurate diagnosis and surgical planning [36].

Once the 3D images are obtained, software tools such as Dolphin and SimPlant are used to generate virtual models of the patient's jaw and skull [37]. Dolphin Imaging is widely used for its comprehensive functionality, allowing surgeons to visualize the skeletal anatomy, plan osteotomies, and simulate the repositioning of bone segments in a three-dimensional space [38]. On the other hand, SimPlant integrates CAD/CAM technology to design patient-specific surgical guides and splints that can be 3D-printed and used intraoperatively to ensure precise bone cuts and accurate jaw positioning. These tools allow surgeons to preview the outcomes of various surgical strategies, enabling them to select the best approach for each patient [36]. The use of CAD/CAM in conjunction with VSP further enhances precision by enabling the design of custom-fit surgical splints, fixation plates, and templates [39].

Clinical Applications

The primary benefit of VSP in orthognathic surgery is the improvement in surgical accuracy [38]. Conventional methods, which rely on 2D imaging and manual planning, often result in deviations between the planned and actual outcomes. VSP, by contrast, allows surgeons to plan osteotomies and jaw repositioning with sub-millimeter precision, reducing the likelihood of complications such as asymmetry or malocclusion [37]. Studies have shown that surgeries planned with VSP result in significantly higher accuracy in achieving the desired postoperative outcomes compared to traditional approaches (Table-1) [39][40]. highlights that VSP generally offers superior clinical outcomes compared to conventional techniques.

Another major advantage of VSP is its ability to provide patient-specific planning. Each patient's anatomy is unique, and the one-size-fits-all approach of traditional methods often fails to account for individual variations in craniofacial structure [42]. VSP enables surgeons to tailor the surgical plan to each patient’s specific needs, ensuring that the surgical strategy aligns with the patient's anatomy and aesthetic goals [36]. This personalized approach leads to better functional and aesthetic results, as well as higher levels of patient satisfaction [35]. VSP also contributes to a reduction in intraoperative risks. By simulating the procedure in advance, surgeons can anticipate potential challenges, such as difficult osteotomies or complex anatomical relationships, and plan accordingly [43]. This proactive approach minimizes the likelihood of complications during surgery, such as nerve damage or excessive blood loss, and can lead to shorter operating times [34][40]. Additionally, the use of pre-designed surgical guides ensures that the actual bone cuts follow the planned paths, reducing the need for intraoperative adjustments and thereby lowering the risk of errors [39,.40]. Numerous studies have confirmed the superior accuracy and predictability of VSP compared to conventional methods [13][33][43]. A study by Stokbro et al. [8] demonstrated that VSP-assisted surgeries achieved a mean discrepancy of less than 1 mm between the planned and postoperative jaw positions, a significant improvement over the 2-3 mm deviations commonly seen in manually planned surgeries. Similarly, studies comparing CAD/CAM-assisted VSP with traditional planning methods have shown that VSP not only improves accuracy but also reduces operating times and the likelihood of surgical revisions [36]. Moreover, Ho et al. [10] evaluated the use of 3D-printed surgical guides and found that the guides allowed for precise execution of osteotomies and ensured better postoperative symmetry, particularly in complex cases. Additionally, meta-analyses have shown that VSP reduces the need for postoperative adjustments, which is a common issue in surgeries performed using traditional methods [13]. The consistent accuracy provided by VSP has led to its growing adoption in the field of orthognathic surgery, where predictable results are crucial for both functional and aesthetic outcomes [44].

Combination of Robotic Assist and VSP

The integration of VSP with robotic systems represents a major leap forward in the field of orthognathic surgery, combining the preoperative precision of VSP with the intraoperative accuracy and control of robotic assistance [6]. This synergy enhances surgical outcomes by minimizing human error, improving precision during complex procedures, and ensuring that the preoperative plans are executed with remarkable fidelity [5]. VSP enables surgeons to map out the entire procedure in a 3D virtual environment, simulating osteotomies, bone movements, and even the final alignment of the mandible and maxilla [33]. Robotic systems allow for the precise execution of these plans during surgery. The key advantage of this integration lies in the robotic system's ability to follow the virtual plan with sub-millimeter accuracy, reducing the variability that can arise from manual execution [3][30]. The typical workflow begins with the VSP phase, where the surgeon uses imaging data (CT or CBCT scans) to construct a detailed virtual model of the patient’s craniofacial anatomy [11]. Once the surgical plan is optimized in the virtual environment including the design of cutting guides, osteotomy paths, and fixation strategies. This data is transferred to the robotic system [42]. The robotic arms can then execute the bone cuts and repositioning movements with extreme precision, following the planned trajectory and ensuring consistency between the virtual plan and the actual surgery [32][45].

Moreover, intraoperative navigation systems integrated with robotics allow for real-time tracking and adjustments based on the patient's anatomy during surgery [32]. This ensures that any intraoperative variations, such as slight shifts in bone position, are accounted for, enabling the robotic system to make fine adjustments based on live feedback [4]. In combination with robotic assistance and VSP for mandible reconstruction by using fibula-free flaps, VSP enables precise preoperative mapping of the fibula to the mandibular defect using CT imaging, defining the ideal osteotomy (bone-cutting) paths [46]. The robotic system then executes these plans with high precision, guided by optical navigation, improving accuracy and reducing human error during the fibular bone cutting and alignment process [26][46]. A clinical trial on 35 patients showed that this system significantly reduced surgery time, enhanced the accuracy of bone cuts, and minimized complications, offering a safer, more efficient approach to mandible reconstruction [46]. Also, Wu et al. [5] demonstrated that robotic systems and VSP integration help to reduce the time needed for manual bone manipulation, improve the accuracy of bone positioning, and provide the potential for better postoperative outcomes, especially in complex cases where conventional methods might fall short. Moreover, Kong et al. showed [47] the effectiveness of a multi-arm robotic system in improving the precision of mandibular reconstruction surgeries. Their system, which integrates robotic assistance with optical navigation, was able to reduce placement errors to as low as 1.02 mm during fibular segment positioning [47]. The experiments conducted on skull models and animal subjects showed that robotic assistance not only minimizes surgeon fatigue but also enhances the accuracy of bone graft alignment compared to manual methods. This aligns with previous studies highlighting the advantages of robotics in improving surgical outcomes by ensuring consistent precision and reducing operative risks [47]. The combination of robotic systems and VSP is also beneficial in revision surgeries, where previous orthognathic surgeries have not achieved the desired outcomes, or complications have arisen [5]. Revision surgeries require extreme precision to correct previous errors without further compromising jaw alignment or function. In such cases, VSP allows for a thorough evaluation of the current anatomy and planning of corrective osteotomies, while robotic systems ensure the execution is precise, minimizing the chance of further complications [5][46].

## Technical Limitation

While the benefits of robotic assistance and VSP in orthognathic surgery are frequently highlighted, it is equally important to address their shortcomings, particularly in complex multi-segment surgeries and during the fixation phase.

VSP in Multi-Segment Surgeries: VSP's precision may falter in multi-segment surgeries, where small misalignments can propagate through subsequent surgical steps [8]. When multiple jaw segments need to be realigned, even minute errors during planning or execution can lead to significant postoperative asymmetry [48]. While VSP improves initial planning, its reliance on static preoperative data may not always adapt well to dynamic intraoperative factors, such as changes in tissue tension or unexpected anatomical variations [49].

Robotic Handling of Fixation

Robotic systems excel at precision cutting, but challenges arise during the fixation phase. For example, Liang et al. [50] found that while robotic systems achieve high precision in maxilla-mandibula complex repositioning, the accuracy during fixation was less satisfactory, with an average holding accuracy of 1.56 ± 1.2 mm. This highlights the difficulty in maintaining precise positioning during the fixation process [50]. The process of placing plates and screws is often more complex, especially in confined areas like the craniofacial region [36]. The variability in bone density and the difficulty of positioning fixation hardware without affecting surrounding tissues often require manual adjustments, reducing the potential for robotic accuracy during this crucial phase [51]. Also, current robotic systems lack sophisticated real-time feedback mechanisms to adjust fixation based on intraoperative changes, increasing the risk of deviations from the surgical plan [46][47]. A study on robotic surgical systems suggested that current platforms still struggle with real-time adaptations during fixation procedures, and the absence of dynamic force feedback further complicates the process [46].

Technical Challenges and System Errors: The reliance on technology introduces the potential for system errors, including software malfunctions, calibration errors, or navigation inaccuracies [5][47]. For instance, Wu et al. [5] identified minor osteotomy errors of around 1.07 ± 0.19 mm due to navigation inaccuracies, which could result in intraoperative deviations that are difficult to correct manually. In addition, surgical navigation systems may not always provide consistent tracking, further complicating procedures where high precision is essential [52].

## Challenges and Barriers

Despite the potential of robotic-assisted surgery and VSP to revolutionize orthognathic surgery, several challenges and barriers hinder widespread adoption. These challenges span from regulatory and cost-related hurdles to cultural resistance within the surgical community and infrastructure limitations [52]. Robotic platforms such as the Da Vinci® Surgical System can cost upwards of $1.5 million US dollars, excluding maintenance, software updates, and additional training costs [53]. Studies have confirmed that the high initial acquisition costs, combined with ongoing service and training expenses, create significant financial hurdles for hospitals, especially smaller institutions or those with lower patient volumes [54]. The long-term cost-benefit of such systems, especially in institutions with lower patient volumes or limited financial resources, is still a subject of debate [55]. Moreover, regulatory approval processes can add complexity to the introduction of new surgical technologies, as they require meeting stringent standards for safety and effectiveness, which can slow down innovation [52][56].

Also, the growing use of artificial intelligence (AI) and VSP in orthognathic surgery introduces significant ethical and data privacy concerns that demand more in-depth discussion [57]. One of the central issues revolves around the use of patient data. AI systems rely heavily on vast datasets, including patient imaging and medical histories, to optimize and refine surgical planning [57][58]. This raises critical issues around patient data ownership and consent, as the use of such data for AI training necessitates robust consent mechanisms to ensure ethical use [58]. Additionally, the responsibility for safeguarding this sensitive information is a growing concern, especially in cases where AI-driven systems may be vulnerable to breaches [59]. Furthermore, in the event of AI-driven errors during surgery, there are critical questions about liability whether the responsibility lies with the surgeon, the software developer, or the institution [60].

Moreover, data privacy concerns in these technologies are paramount. As VSP and robotic systems handle sensitive personal health information, they are susceptible to cybersecurity breaches, which could expose patient identities or medical details, undermining patient trust [61].

Compliance with regulations is crucial, but the ongoing need for large datasets for AI training increases the complexity of maintaining privacy and security [58][61].

Moreover, algorithmic bias presents another ethical dilemma. If AI systems are trained on unrepresentative data, they could inadvertently perpetuate healthcare disparities, leading to unequal surgical outcomes [62]. Ensuring transparency in AI algorithms and safeguarding against biases is essential to ensure fair, unbiased, and secure use of these emerging technologies in surgical practices [61][62].

Moreover, a significant barrier to the adoption of robotic surgery and VSP is cultural resistance within the surgical community [52][56]. Many experienced surgeons who have spent years honing their skills using traditional methods may be hesitant to transition to robotic systems, viewing them as unnecessary or disruptive to their workflow [56].

Additionally, some argue that traditional methods are adequate for achieving desired surgical outcomes, and there is a belief among some practitioners that robotic systems do not necessarily offer significant clinical benefits to justify the investment [56][58].

Furthermore, the steep learning curve in robotic-assisted surgery, particularly with robotic systems, presents a significant barrier to adoption due to the need for extensive training and adaptation [52][56]. Surgeons often require 50-100 cases to gain proficiency, primarily because of the lack of tactile feedback, which forces reliance on visual cues during procedures [56]. To overcome this, solutions such as simulation-based training programs, mentorship from experienced robotic surgeons, and a gradual introduction of robotic systems into surgical practice have been proposed [63]. These strategies help surgeons build competence incrementally, ensuring patient safety while reducing the time and effort needed to master robotic surgery [56][64].

Moreover, there is currently no standardized training protocol, leading to inconsistent skill levels among practitioners [64]. For hospitals, the adoption of robotic-assisted systems also requires substantial infrastructure upgrades, including reconfiguring operating rooms to accommodate the larger footprint of robotic systems and integrating VSP software with hospital information systems [65].

## Prospective Outlook

The future of orthognathic surgery is being shaped by several promising trends, particularly the integration of AI, augmented reality (AR), and ML, which aim to enhance surgical planning and execution. These emerging technologies hold great potential to complement the already impactful synergy of robotic assistance and VSP [66][67].

AI and ML are rapidly transforming surgical planning, especially in areas like image analysis and prediction of surgical outcomes. AI-driven tools can assist in the automation of 3D imaging, creating detailed anatomical models that reduce human error and improve the efficiency of preoperative planning [68][69]. For example, ML algorithms can analyze vast amounts of data from previous surgeries to predict facial morphology changes post-surgery, helping surgeons plan more accurate and personalized procedures [70]. Also, these systems can refine surgical decision-making by learning from patterns in previous cases, improving the accuracy of jaw repositioning and reducing the likelihood of postoperative complications [71].

Studies have demonstrated that AI can optimize skeletal alignments and enhance communication with patients through improved visualization tools [69]. The continued development of AI-based prediction models could lead to more precise virtual simulations that not only guide robotic systems but also adjust intraoperative decisions in real-time, increasing the adaptability of procedures to intraoperative findings. [72].

Moreover, AR is another rapidly evolving technology that has potential applications in intraoperative guidance [66]. By overlaying digital images onto the surgeon’s field of view, AR can provide real-time visual feedback during surgeries, allowing for more accurate osteotomies and precise bone repositioning [73].

This real-time interaction between virtual plans and the physical anatomy is particularly beneficial in complex maxillary or mandibular reconstructions, where traditional visualization methods may fall short [72][74]. AR-guided navigation's dynamic adjustment capabilities can further improve precision and decrease the need for corrective surgeries [74].

Another area of growth is the development of next-generation robotic arms with increased flexibility and real-time navigational adjustments [75]. These systems will enable surgeons to make immediate adjustments based on intraoperative conditions without the need to switch between manual and robotic techniques [75][76]. The future could also see the rise of minimally invasive robotic techniques for orthognathic surgery, reducing recovery times and improving patient comfort [76].

To fully unlock the potential of these technologies, further clinical trials are necessary to validate the long-term benefits of integrating AI, AR, and robotic assistance in orthognathic surgery. By staying at the forefront of these innovations, the field of orthognathic surgery can continue to evolve, offering patients increasingly refined, safe, and effective treatment options [73].

## Conclusion

The integration of robotic assistance and VSP in orthognathic surgery represents a significant advancement in the precision and predictability of surgical outcomes. These technologies have proven to improve osteotomy accuracy, reduce operative times, and enhance patient satisfaction by facilitating personalized, detailed preoperative planning.

Studies consistently show that VSP, combined with robotic systems, reduces the margin of error in bone repositioning and minimizes the likelihood of postoperative complications, which are often present in traditional methods.

From a clinical relevance perspective, the shift towards these technologies is pivotal in improving both functional and aesthetic outcomes. By allowing surgeons to visualize complex craniofacial structures in 3D, simulate surgical outcomes, and execute precise cuts with robotic assistance, the combined use of VSP and robotics leads to better patient-specific care. However, the current barriers, such as high costs, training challenges, and infrastructure requirements, must be addressed to facilitate widespread adoption.

Looking ahead, the potential of AI, AR, and ML to further refine both VSP and robotic assistance offers exciting prospects for the future of orthognathic surgery. These technologies could revolutionize the field by improving real-time decision-making during surgery and further increasing the accuracy of patient outcomes. As innovations continue to emerge Future research should focus on long-term clinical studies and cost-effectiveness analyses to validate the full potential and viability of these technologies. Ultimately, with continued development and integration, these advances have the potential to transform orthognathic surgery, making it safer, more efficient, and more effective for patients worldwide.

## Conflict of Interest

None.
